# Supplementary material for: Comparison of mRNA Vaccinations with BNT162b2 or mRNA-1273 in Anti-CD20-Treated Multiple Sclerosis Patients
Source: Vaccines (Basel). 2022 Jun 9;10(6):922. doi: 10.3390/vaccines10060922 (PMC9229998; doi:10.3390/vaccines10060922)
Supplement: Supplementary file 1 [file vaccines-10-00922-s001.zip › vaccines-1696239-supplementary.pdf]

Table S1. Characteristics of MS patients treated with anti-CD20 drugs (Ocrelizumab, Rituximab). Continuous variables are displayed as the mean and 95% confidence interval; nominal variables are given as numbers and frequencies. AU: arbitrary units; CI: confidence interval; mL: milliliter; MS: multiple sclerosis; *n*: sample size; n.s.: not significant, defined as *p*-values ≥ 0.05 (comment borderline *p*-value center 0.051); PPMS: primary progressive multiple sclerosis; RMS: relapsing MS.

Variables	Total (*n* = 74)	BNT162b2 (*n* = 36)	mRNA-1273 (*n* = 38)	*p*-Value
**Demographic Characteristics**				
Sex (female, n (%))	41 (55.4)	17 (47.2)	24 (63.2)	n.s.
Age (years, mean (95% CI))	46.6 (43.4–49.9)	45.4 (40.4–50.4)	47.8 (43.4–52.3)	n.s.
**Anti-CD20**				
Rituximab	9 (12.7)	7 (19.4)	2 (5.3)	n.s.
Ocrelizumab	65 (87.8)	29 (80.6)	36 (97.4)	n.s.
**Center**				
Bern	26 (35.1)	17 (47.2)	9 (23.7)	n.s.
Lucerne	48 (64.9)	19 (52.8)	29 (76.3)	n.s.
**Diagnosis**				
RMS	57 (77.0)	30 (83.3)	27 (71.0)	n.s.
PPMS	17 (23.0)	6 (16.7)	11 (28.9)	n.s.
**Vaccination**				
Anti-spike IgG (AU/mL)	28.0 (8.6–47.4)	25.6 (−1.1–52.3)	30.2 (0.8–59.6)	n.s.
Time between vaccination and sampling (years)	0.35 (0.3–0.4)	0.41 (0.4–0.5)	0.29 (0.3–0.3)	**0.001**
Time between last dosage and sampling (years)	0.44 (0.4–0.50)	0.51 (0.4–0.6)	0.38 (0.3–0.4)	n.s.

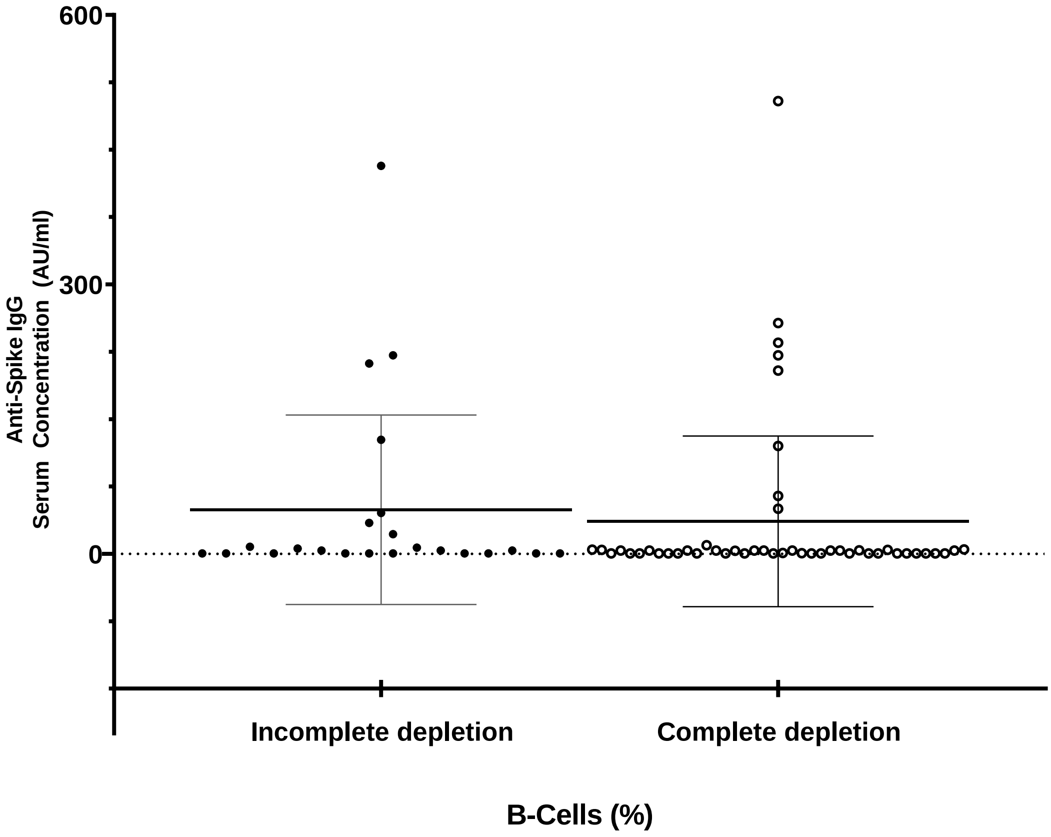

Figure S1. B-lymphocytes (%) of our cohort of anti-CD20-treated patients. AU: arbitrary units; mL: milliliter.
